# Effect of Temperature on Metronidazole Resistance in *Helicobacter pylori*

**DOI:** 10.3389/fmicb.2021.681911

**Published:** 2021-05-19

**Authors:** Meiliang Gong, Yingjie Han, Xuning Wang, Hongjin Tao, Fansen Meng, Baicun Hou, Benjamin B. Sun, Gangshi Wang

**Affiliations:** ^1^Department of Laboratory Medicine, Second Medical Center, Chinese PLA General Hospital, Beijing, China; ^2^Department of Gastroenterology, Second Medical Center, Chinese PLA General Hospital, National Clinical Research Center for Geriatric Diseases, Beijing, China; ^3^Department of Oncology, Fifth Medical Center, Chinese PLA General Hospital, Beijing, China; ^4^Department of Surgery, First Medical Center, Chinese PLA General Hospital, Beijing, China; ^5^British Heart Foundation Cardiovascular Epidemiology Unit, Department of Public Health and Primary Care, University of Cambridge, Cambridge, United Kingdom; ^6^Royal Free Hospital, London, United Kingdom

**Keywords:** *Helicobacter pylori*, temperature, transcriptomics, antibiotic resistance, metronidazole

## Abstract

Efficacy of *Helicobacter pylori (H. pylori)* eradication therapy has declined due to rapid rises in antibiotic resistance. We investigated how increased temperature affected *H. pylori* (NCTC 11637) growth and its sensitivity to metronidazole *in vitro*. We performed transcriptomic profiling using RNA-sequencing to identify differentially expressed genes (DEGs) associated with increased temperature. Transcriptional pathways involved in temperature-driven metronidazole resistance changes were analyzed through bioinformatic and literature curation approaches. We showed that *H. pylori* growth was inhibited at 41°C and inhibition was more apparent with prolonged incubation. Resistance to metronidazole was also reduced—minimum inhibitory concentration for metronidazole decreased from > 256 μg/ml at 37°C to 8 μg/ml at 41°C after culturing for 3 days. RNA-sequencing results, which were highly concordant within treatment conditions, revealed more than one third of genes (583/1,552) to be differentially expressed at increased temperatures with similar proportions up and down-regulated. Quantitative real-time PCR validation for 8 out of 10 DEGs tested gave consistent direction in gene expression changes. We found enrichment for redox and oxygen radical pathways, highlighting a mechanistic pathway driving temperature-related metronidazole resistance. Independent literature review of published genes associated with metronidazole resistance revealed 46 gene candidates, 21 of which showed differential expression and 7 out of 9 DEGs associated with “redox” resistance pathways. Sanger sequencing did not detect any changes in genetic sequences for known resistance genes *rdxA, frxA* nor *fdxB.* Our findings suggest that temperature increase can inhibit the growth and reduce *H. pylori* resistance to metronidazole. Redox pathways are possible potential drivers in metronidazole resistance change induced by temperature. Our study provides insight into potential novel approaches in treating antibiotic resistant *H. pylori*.

## Introduction

*Helicobacter pylori (H. pylori)* infection has been established as the main cause of various gastroduodenal diseases including chronic gastric inflammation, peptic ulcer disease and gastric cancer (GC) and it is classified as a class I carcinogen (Ishaq and Nunn, 2015). *H. pylori* resides in human gastric mucosa and affects nearly half of the human population worldwide with prevalence exceeding 80% in certain regions such as parts of Asia (Hooi et al., 2017). Eradication of *H. pylori* has been shown to reduce GC incidence across a range of risk groups (Takenaka et al., 2007; Kosunen et al., 2011).

The standard triple therapy, consisting of a proton pump inhibitor combined with clarithromycin and amoxicillin or metronidazole, has been the mainstay of treatment for *H. pylori* infection over the last two decades. However, rising antibiotic resistance has made standard triple therapy less effective—bismuth and non-bismuth based quadruple therapies have also been introduced depending on efficacy and resistance patterns locally (Graham and Fischbach, 2010). Resistance to clarithromycin and metronidazole also varies between regions across the world and have hampered the elimination of *H. pylori* (Kim et al., 2015). In China, the resistance rate for clarithromycin is around 50% whilst resistance to metronidazole ranges from 41.6 to 95.4% in Southeast China (Su et al., 2013; Thung et al., 2016). Various mechanisms have been shown to affect *H. pylori* antibiotic resistance under physiological conditions (Hu et al., 2016; Alba et al., 2017). Point mutations in 23S rRNA and changes in efflux pump systems have been shown to confer resistance to macrolides such as clarithromycin (Francesco et al., 2011). In addition, reduced activities in nitro-reductase (rdxA), flavinoxido reductase (frxA) and ferrodoxin-like enzymes (frxB) lead to reduced activation of metronidazole (Francesco et al., 2011).

Changes in environmental conditions lead to alterations in transcriptomic profiles of *H. pylori* (Thompson et al., 2003; De la Cruz et al., 2017). Previous evidence has demonstrated local nanoparticle heating inhibits *H. pylori* growth and virulence as a potential alternate approach to treating *H. pylori* infections (Wu et al., 2019; Zhi et al., 2019). Whilst temperature differences have led to changes leading to increased antibiotic resistance in other bacteria species (Liang et al., 2016; Loughman et al., 2016; De Silva et al., 2018), the effects of temperature on antibiotic resistance and transcriptomic changes in *H. pylori* have not been investigated previously.

In this study, we examine the effect of changes in temperature on the growth of *H. pylori* and its sensitivity to metronidazole using a reference resistant strain. In addition, we investigate perturbations in transcriptomic profiles underlying changes in temperature-driven antibiotic susceptibility.

## Materials and Methods

### Bacterial Strain and Culture Conditions

*H. pylori* type strain NCTC 11637 (Lock et al., 2001) was used for this study and 16S rRNA identification was used to verify the strains. Bacteria was cultured on Karmali Agar Base (CM0935, Oxoid, United Kingdom) supplemented with 5% sterile defibrinated sheep blood (Beijing XLF Medical Sales Co. Ltd, China) for 3–5 days at 37°C, under microaerophilic conditions: microaerobic gas mixture composed of 5% oxygen, 10% carbon dioxide, and 85% nitrogen (GEN bag microaer, BioMérieux, France). *H. pylori* were subcultured three times before each experiment.

### Comparison of *H. pylori* Growth at Different Temperatures

*H. pylori* NCTC 11637 was cultured at 37 and 41°C to evaluate the effects of elevated temperature on bacteria growth. Bacteria was resuspended in phosphate-buffered saline (PBS) to 8 different dilutional concentrations (0.5, 0.25, 0.125, 0.0625, 0.03125, 0.015, 0.0075, 0.003 McFarland). 90 mm plates seeded with 0.1 ml of bacterial suspensions were incubated at 37°C as controls. In the treatment group, 0.1 ml bacterial suspensions at the 8 dilution concentrations were seeded on 3 sets of 8 plates. After inoculation, the 3 sets of plates (each set consists of 8 plates of different bacterial concentrations) were incubated at 41°C for 1, 3, and 5 days, respectively, and then incubated at 37°C for a further 3 days. Colonies in each plate were counted after the last incubation.

### Antibiotics Susceptibility Testing

*In vitro* minimum inhibitory concentrations (MICs) of four antibiotics (amoxicillin, clarithromycin, metronidazole, tetracycline) against *H. pylori* NCTC 11637 were tested. All experiments were performed in triplicate. The MICs of amoxicillin, metronidazole and tetracycline against *H. pylori* were determined via the Epsilometer test (E-test) using an E-strip (BioMerieux SA, France) and the Kirby-Bauer method was used for clarithromycin sensitivity. *H. pylori* NCTC 11637 was cultured as described above, and the third-generation colonies were selected and suspended in PBS to the turbidity of a 2 McFarland standard. Then, 0.1 ml of the bacterial suspension was evenly coated on Karmali Agar Base. Each agar plate was left to dry for 15 min before E-strip was affixed and the plates were incubated as described above. MICs were defined as the lowest concentration that allowed no visible growth after 72 h of incubation at 37°C. The clinical breakpoints for amoxicillin, clarithromycin, metronidazole, and tetracycline are defined as: >0.125, >0.5, >8, and >1 mg/L, respectively, as per European Committee on Antimicrobial Susceptibility Testing Breakpoints version 8 (European Committee on Antimicrobial Susceptibility Testing [ECUCAST], 2018).

### RNA Extraction and Transcriptomic Analysis

*H. pylori* NCTC 11637 strain was divided into two groups in triplicate. The experimental group was treated at 41°C for 3 days. The control group was treated at 37°C for 3 days. The cells were harvested, and total RNA was extracted and purified using the Bacterial RNA kit (Omega Bio-tek, GA, United States) according to manufacturer protocols. Quality control of each RNA sample was performed with Agilent 2100 Bioanalyzer (Agilent Technologies, Beijing, China). The cDNA libraries were constructed using NEBNext^®^ Ultra^TM^ RNA Library Prep Kit (New England Biolabs, Ipswich, MA, United States) and submitted for sequencing using IlluminaHiseq 4000. Library construction and sequencing were performed by Allwegene BioTech Co., Ltd. (Beijing, China). Raw reads were filtered using Trimmomatic v0.33 (Bolger et al., 2014) and mapped to the *H. pylori* NCTC 11637 genome (National Center for Biotechnology Information [NCBI], 2012) using Bowtie2 v2.2.6 (Langmead and Salzberg, 2012) with default parameters. Genes were quantified using HTSeq v0.6.0 (Anders et al., 2015). Differentially expressed genes (DEGs) were analyzed by DESeq2 v1.22.1 in R (Love et al., 2014). Genes with absolute log_2_ fold-changes > 1 and multiple testing adjusted (Benjamini–Hochberg procedure) *q* < 0.05 were considered as DEGs.

### Gene Expression Using qRT-PCR

Quantitative real-time polymerase chain reaction (qRT-PCR) assays were performed using the same samples analyzed (3 controls at 37°C and 3 at 41°C for 3 days) as in the RNA-seq transcriptomic analyses. 10 genes with differential expression levels identified using RNA sequencing were selected for subsequent validation (5 of the most significantly associated genes and 5 associated with metronidazole resistance from bioinformatic screening as described in *Curation of resistance related genes*). Gene-specific primers (Supplementary Table 1A) were designed and purchased from Invitrogen (Beijing, China). Three technical replicates were performed for each biological sample. First strand cDNA was synthesized using PrimeScript^TM^ RT reagent Kit with gDNA Eraser (Takara, Japan) according to manufacturer instructions. qRT-PCR reactions were performed using an ABI Prism 7500 Sequence Detection System (Perkin-Elmer Applied Biosystems, Foster City, CA). *H. pylori* 16S rRNA was used as housekeeping internal control. Gene expression and log_2_ fold-changes were analyzed using the 2^–ΔΔ*CT*^ algorithm (Livak and Schmittgen, 2001). After quality control, one biological sample at 37°C exhibited low readouts across all genes compared to the other 2 biological samples (which did not show low readouts in RNA-seq) and was excluded from analysis.

### GO and KEGG Pathway Analysis

We mapped the genes to Entrez Gene symbols first. Gene Ontology (GO) enrichment analysis comprising cellular component (CC), molecular function (MF), and biological process (BP) were conducted for DEGs in R with GOSeq v1.26.0 and topGO v2.26.0 package, using the *H. pylori* 11637 reference strain annotated by Pfam (protein families database) (El-Gebali et al., 2019) as background. KOBAS v3.0 (Xie et al., 2011) was used to perform Kyoto Encyclopedia of Genes and Genomes (KEGG) pathway enrichment analysis for DEGs using *H. pylori* 26695 reference strain as background. The *p* < 0.05 adjusted for multiple testing (*q*-value) using the Benjamini-Hochberg method was used as significance thresholds for GO and KEGG pathway enrichment analyses. We also performed sensitivity analyses using absolute log_2_ fold-change > 1.5, *q* < 0.05 as cut-offs for DEGs.

### Curation of Resistance Related Genes

#### Curation of All Mapped Genes Into Metronidazole Resistance Pathways

Metronidazole antibiotic resistance pathways in *H. pylori* based on four groups of broad mechanisms: (1) reduced activity of nitro-reductases, (2) increased activity of the oxygen radical scavenger system, (3) reduced uptake and increased efflux, and (4) increased activity of the DNA repair enzymes, were determined from current literature (Hu et al., 2016; Alba et al., 2017). All mapped genes (*n* = 1,552) were manually curated by two independent reviewers to determine whether each is related to one of the four mechanisms based on gene function and gene description. Genes with assignments which agree between the two reviewers were included.

#### Curation of Genes Associated With Metronidazole Resistance in Literature

Queries were made in PubMed using keywords “helicobacter pylori,” “metronidazole,” and “resist^∗^” and subsequently manually curated to create a list of reported *H. pylori* metronidazole resistance genes. DEGs identified in this study were then mapped to the list of literature reported genes. Enrichment tests were performed by permutation testing (10,000 times).

### Detection and Sequencing of rdxA, frxA, and fdxB

For both 37°C and 41°C conditions, conventional polymerase chain reaction (PCR) amplification was performed. Specific reagents, primers and conditions are detailed in Supplementary Table 1B. PCR, gel electrophoresis and DNA sequencing via Sanger sequencing method were performed following standard manufacturer protocols.

## Results

### Increased Temperature Inhibits *H. pylori* Growth

**FIGURE 1:**
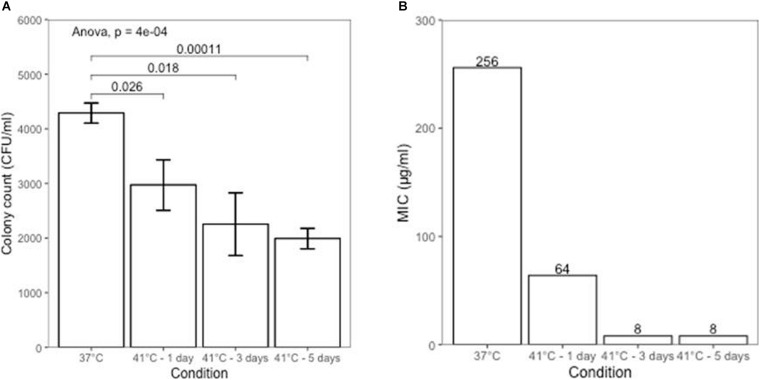
**(A)** Growth inhibition of *H. pylori* at different temperature conditions. **(B)** Changes in metronidazole susceptibility at different temperature conditions.

The effect of elevated temperature on *H. pylori* (NCTC 11637 strain) growth was evaluated at 37°C (control group) and at 41°C for 1, 3, and 5 days. Growth of *H. pylori* was significantly inhibited after incubation at 41°C with inhibition more apparent with prolonged incubation (Figure 1A). At an inoculation concentration of 0.002 McFarland, bacterial colony count of the control group was 4,288 ± 184 CFU/ml. The counts decreased to 2,970 ± 462, 2,255 ± 575, and 1,990 ± 187 CFU/ml after 1, 3, and 5 days of treatment under 41°C, respectively (*p* = 0.026 [1 day], *p* = 0.018 [3 days], *p* = 0.00011 [5 days], compared with control group).

### Temperature Change Increases Sensitivity of *H. pylori* to Metronidazole

We investigated the effect of increased temperature on sensitivities of the metronidazole-resistant *H. pylori* NCTC 11637 strain to metronidazole using the E-test strip. The minimum inhibitory concentration (MIC) for metronidazole under the 37°C culture condition (control group) was > 256 μg/ml. After culturing at 41°C for 3 days, MIC of *H. pylori* to metronidazole decreased to 8 μg/ml (Figure 1B), which is the breakpoint of metronidazole resistance. The NCTC 11637 strain was sensitive to clarithromycin, amoxicillin and tetracycline and this did not change under increased culture temperature conditions (Supplementary Table 2). There was no growth in the *H. pylori* subculture inoculation after returning from 41 to 37°C.

### Transcriptome Analyses Identify Changes in Drug Resistance Genes

#### Identification of Differentially Expressed Genes by RNA-Sequencing

**FIGURE 2:**
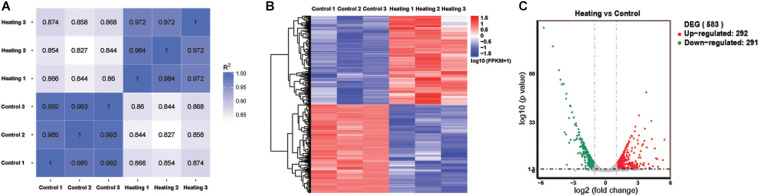
Differentially expressed genes (DEGs) by RNA-seq. **(A)** Pearson’s correlation heatmap of expression of all mapped genes **(B)** Hierarchical clustered heat map of significantly differentially expressed genes (583). **(C)** Volcano plot displaying DEGs. The vertical axis corresponds to the *q*-value, the horizontal axis displays the log_2_ fold-change. The red dots represent the up-regulated expressed transcripts (292), the green dots represent the down-regulated transcripts (291). Vertical and horizontal dashed lines indicate absolute log_2_ fold-change = 1 and adjusted *p* = 0.05, respectively.

To study the transcriptomic changes which may drive decreased resistance to increased temperature, we used RNA sequencing to assess changes in gene expression between 37 and 41°C. Illumina paired-end sequencing of 6 samples (3 cultured at 37°C and 3 at 41°C) yielded a total of 252,222,378 clean reads. 70.9–80.1% reads of samples were mapped to the annotated *H. pylori* NCTC 11637 genome culminating in 1,552 mapped genes. Gene expression measurements were highly consistent between biological replicates within each temperature condition (Pearson’s *r*: 0.97–0.99 within condition, Figure 2A). 583 out of 1,552 mapped genes were significantly differentially expressed at absolute log_2_ fold-change > 1 and *q* < 0.05 after incubation at 41°C for 3 days compared to 37°C, of which 292 were up-regulated and 291 were down-regulated (Figures 2B,C and Supplementary Table 3).

#### Gene Expression Measurements Using qRT-PCR

**FIGURE 3:**
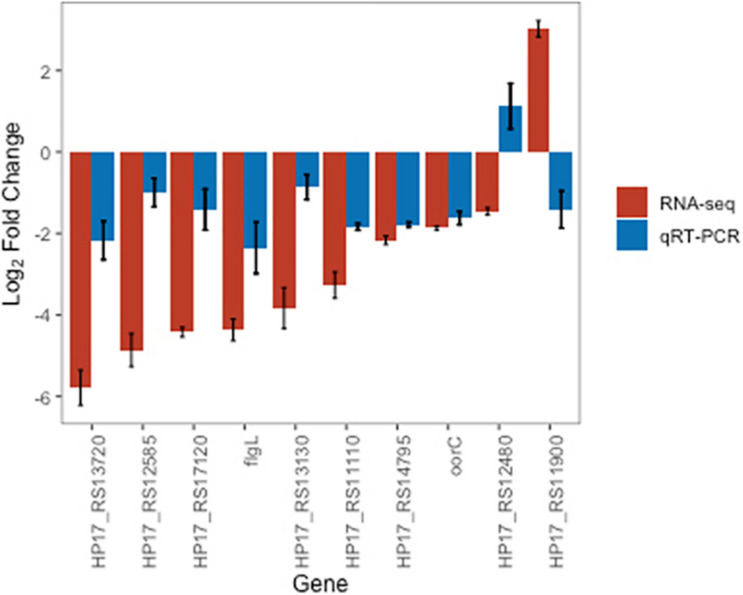
Barplot showing log_2_ fold-changes at incubation of 41°C for 3 days compared to 37°C using RNA-Seq and qRT-PCR methods for 10 selected DEGs. Error bars indicate standard deviations from the mean. Genes ordered by RNA-Seq log_2_ fold-changes.

We selected 10 DEGs, 5 most significantly associated genes and 5 related to metronidazole resistance (Materials and Methods) for measurement using qRT-PCR. 8 out of the 10 DEGs showed consistent direction in log fold gene expression changes (Figure 3). All 5 of DEGs with the strongest statistical significance of association [HP17_RS13720 (HP1076), HP_RS12585 (HP0115), flgL, HP17_RS17120 (HP0630), and HP17_RS13130 (HP1286)] showed directionally concordant changes using qRT-PCR.

#### GO and KEGG Pathway Enrichment Analysis

To investigate molecular and functional pathway changes as result of increased temperature, GO and KEGG pathway enrichment analyses of DEGs were performed. Whilst no pathways were significantly enriched after adjusting for multiple testing, we found enrichment at nominal significance (*p* < 0.05) for drug, vitamin, superoxide and reactive oxygen species metabolic GO processes. Cellular component was enriched for ribosome, non-membrane-bounded organelle and cytoplasm components at nominal threshold. Molecular function enrichment included structural constituent of ribosome, oxidoreductase activity, superoxide dismutase activity and various symporter activities (Supplementary Tables 4.1, 4.2). Sensitivity analyses, using DEGs defined using more stringent thresholds of absolute log_2_ fold-change > 1.5 adjusted *q* < 0.05 showed similar results. Both analyses showed GO enrichment for reactive oxygen species and metabolic processes (Supplementary Table 5).

### DEGs Are Enriched for Specific Metronidazole Resistance Pathways

All mapped genes were curated base on gene description and function into four broad metronidazole resistance pathways: (1) nitro-reductases, (2) oxygen radical scavenger, (3) drug uptake and efflux and (4) DNA repair related. 126 genes were related to one of the four pathways (Supplementary Table 6 and Table 1). Nitro-reductase related pathways were enriched for DEGs under increased temperature condition at 41°C (permutation 10,000 times, empirical *p* = 0.0038). Oxygen radical scavenger pathways were borderline significant for enrichment of DEGs (empirical *p* = 0.05).

**TABLE 1:** Curation of DEGs into metronidazole resistance pathways (adjusted *p* < 0.05).

**Resistance mechanism**	**Gene**	**Gene description**	**Log_2_ (fold change)**	**Adjusted *p*-value**
DNA repair (12/32 genes, enrichment *p* = 0.57)	HP17_RS15190	DNA (cytosine-5-)-methyltransferase	2.737	8.89E–31
	HP17_RS17710	DNA cytosine methyltransferase	2.476	7.30E–05
	HP17_RS14395	DNA polymerase III subunit delta’	2.209	1.39E–11
	HP17_RS13655	DNA translocase FtsK	–2.026	6.78E–18
	HP17_RS10090	DNA gyrase subunit A	–1.928	1.28E–16
	HP17_RS10700	DNA-binding response regulator	–1.904	5.06E–13
	HP17_RS12590	DNA topoisomerase I	1.893	1.46E–16
	HP17_RS15830	Thymidylate synthase (FAD)	–1.602	2.12E–11
	HP17_RS10880	DNA (cytosine-5-)-methyltransferase	1.473	7.16E–10
	HP17_RS14590	DNA-directed RNA polymerase subunit beta/beta’	1.174	2.01E–07
	HP17_RS16580	DNA starvation/stationary phase protection protein	–1.151	1.86E–07
	HP17_RS15450	DNA polymerase III subunit alpha	–1.045	1.11E–05
Drug transportation (25/78 genes, enrichment *p* = 0.88)	HP17_RS11900	Glucose/galactose MFS transporter	3.067	7.56E–25
	HP17_RS13150	Nicotinamide riboside transporter PnuC	3.043	6.80E–10
	HP17_RS11910	Glutamine ABC transporter substrate-binding protein	–2.846	3.78E–32
	HP17_RS16625	ABC transporter permease	2.690	1.28E–27
	HP17_RS13650	MFS transporter	–2.222	2.78E–17
	HP17_RS15125	Iron chelating transport ATP-binding protein	–2.040	1.09E–16
	HP17_RS14795	MFS transporter	–1.935	2.03E–10
	HP17_RS15130	Iron chelating ABC transporter permease	–1.932	5.41E–08
	HP17_RS16620	ABC transporter ATP-binding protein	1.922	1.14E–07
	HP17_RS10180	Lysine transporter	–1.874	9.77E–03
	HP17_RS16500	SulP family inorganic anion transporter	1.850	1.48E–02
	HP17_RS16985	MFS transporter	–1.794	1.49E–14
	HP17_RS16850	Autotransporter domain-containing protein	–1.754	1.86E–12
	HP17_RS17445	AI-2E family transporter	–1.582	6.10E–06
	HP17_RS15475	ABC transporter ATP-binding protein	–1.563	1.09E–06
	HP17_RS17915	ABC transporter ATP-binding protein	1.527	1.71E–05
	HP17_RS16110	ABC transporter substrate-binding protein	1.514	1.10E–06
	HP17_RS14930	ABC transporter ATP-binding protein	–1.473	9.38E–09
	HP17_RS09990	Ferrous iron transport protein B	–1.433	2.47E–09
	HP17_RS15230	Biopolymer transporter ExbD	1.261	7.33E–08
	HP17_RS11620	MATE family efflux transporter	1.222	1.01E–02
	HP17_RS13700	ABC transporter ATP-binding protein	1.139	8.19E–07
	HP17_RS14700	Molybdenum ABC transporter ATP-binding protein	–1.129	1.81E–03
	HP17_RS11565	ABC transporter ATP-binding protein	1.068	9.17E–06
	HP17_RS14520	ABC transporter ATP-binding protein	–1.009	1.74E–05
Nitro-reductase (10/13 genes, enrichment *p* = 0.0038)	HP17_RS17120	Flavodoxin family protein	–4.185	1.30E–62
	HP17_RS17325	2-oxoglutarate ferredoxin oxidoreductase subunit beta	–1.851	3.38E–16
	oorC	2-oxoglutarate:acceptor oxidoreductase	–1.849	1.07E–15
	HP17_RS11960	Flavodoxin	–1.664	9.96E–14
	HP17_RS15890	Pyruvate ferredoxin oxidoreductase subunit beta	–1.651	9.16E–14
	HP17_RS17330	2-oxoglutarate synthase subunit alpha	–1.632	3.93E–13
	HP17_RS12480	NAD(P)H-dependent oxidoreductase	–1.512	3.79E–05
	porC	Pyruvate flavodoxin oxidoreductase subunit gamma	–1.345	8.80E–08
	HP17_RS15705	Cytochrome c oxidase accessory protein CcoG	–1.239	6.23E–08
	HP17_RS15895	2-ketoisovalerate ferredoxin oxidoreductase subunit alpha	–1.161	1.68E–06
Oxygen-radical scavenging (3/3 genes, enrichment *p* = 0.05)	HP17_RS11110	Superoxide dismutase	–3.293	4.11E–45
	HP17_RS17265	3-methyladenine DNA glycosylase	1.904	1.31E–05
	HP17_RS13135	Thiaminase II	1.643	3.72E–04

In addition, we performed independent literature search for published genes associated with metronidazole resistance. Our literature search revealed 46 gene candidates related to metronidazole resistance in *H. pylori* which were classified into 10 categories (Supplementary Table 7). These genes were all found within the 1,552 genes sequenced in our study. 21 of the 46 genes showed differential expression. Permutation testing did not reveal significant enrichment amongst resistance genes overall for DEGs (empirical *p* = 0.15). However, genes associated with “redox” resistance pathways were significantly enriched for DEGs with 7 out of 9 genes differentially expressed (empirical *p* = 0.016). Other pathways with more than 5 genes in each category did not show enrichment for DEGs.

Metronidazole is a prodrug which is first activated by nitro-reductase (mutated in resistant *H. pylori* NCTC 11637 strains; Debets-Ossenkopp et al., 1999) to produce oxygen radicals toxic to bacteria through DNA damage (Cederbrant et al., 1992; van der Wouden et al., 2001). We found that expression of NAD(P)H-dependent oxidoreductase (*rdxA [HP17_RS12480])* was downregulated (log_2_ fold-change: −1.51, *p* = 3.8 × 10^–5^). In addition, we also found that superoxidase dismutase (*Sod [HP17_RS11110]*) expression, an important protein in detoxifying free oxygen radicals, was downregulated (log_2_ fold-change: −3.29, *p* = 4.1 × 10^–45^) and expression of its transcriptional repressor, ferric uptake regulator (*Fur* [HP17_RS13980]) (Tsugawa et al., 2011), was upregulated (log_2_ fold-change: 2.14, *p* = 1.7 × 10^–8^).

### Key Resistance Gene Sequences Not Altered by Increased Temperature

We independently tested established *H. pylori* resistance genes *rdxA, frxA*, and *fdxB* through Sanger sequencing to see if increased temperature conditions led to structural changes in these genes. All three genes were successfully detected and amplified though PCR in both temperature conditions (Supplementary Figure 1). Sanger sequencing did not detect any changes in genetic sequences for *rdxA, frxA* nor *fdxB* (Supplementary Figures 2–Supplementary Figures 4).

## Discussion

Our study showed that increased temperature inhibited the growth of metronidazole resistant *H. pylori* strain (NCTC 11637) consistent with previous reported changes in growth of sensitive *H. pylori* strains (26695 strain) under elevated temperatures (Jiang and Doyle, 1998). In addition, we present the novel finding that elevated temperature (41°C) increased sensitivities of the resistant strain to metronidazole which has not been reported previously. Studies have identified thermoregulated antibiotic resistance in some species. *Francisella tularensis* showed increased resistance to gentamicin at ambient temperature (26°C) compared to mammalian body temperature (37°C) (Loughman et al., 2016). Temperature associated expression changes in antibiotic resistance were observed in *Acinetobacter baumannii* ATCC 17978 (De Silva et al., 2018).

This is the first study investigating gene expression changes with increased temperature in *H. pylori*. Transcriptomic profiling showed good internal consistency within treatment conditions and widespread changes in gene expression with more than one third of all genes differentially expressed. Previous literature showed transcriptomic changes in *H. pylori* with sudden decreased temperature (Han et al., 2009). Approximately equal proportion of DEGs was up and down-regulated similar to findings seen with decreased temperature.

Whilst no specific GO or KEGG pathways were enriched for DEGs after multiple testing correction, we found nominal statistical evidence indicating that metabolic processes may be enriched. Furthermore, permutation analyses based on annotation of genes for metronidazole resistance mechanisms and specific metronidazole resistance genes published in the literature showed enrichment for redox (oxygen radical scavenger) and nitro-reductase pathways. Metronidazole is a prodrug which is first activated by nitro-reductase to produce oxygen radicals toxic to bacteria through DNA damage (van der Wouden et al., 2001). Increased nitro-reductase activity in theory leads to reduced metronidazole resistance. We found reduced expression of nitro-reductases, such as NAD(P)H-dependent oxidoreductase (*rdxA*, *HP17_RS12480)* with heating. The *H. pylori* NCTC 11637 strain used in this study contains a transposon-induced deletion in *rdxA* which plays a key role conferring resistance (Debets-Ossenkopp et al., 1999), thus the NAD(P)H-dependent oxidoreductase produced by the mutated gene does not provide normal functions regardless of expression levels.

In addition we also found *Sod* expression to be downregulated with elevated temperature. Sod contributes to metronidazole resistance through mitigating oxygen radical related damage and increased Sod levels have been associated with development of metronidazole resistance (Wassmann and Bruchhaus, 2000; van der Wouden et al., 2001). Interestingly, we observed an increased expression of a negative transcription regulator of *Sod*, *Fur*, which may also contribute to decreased *Sod* expression. Therefore, another plausible mechanism by which higher temperature leads to reduced metronidazole resistance may be through decreased *Sod* expression which leads to increased susceptibility to superoxide damage. DEGs within other resistance pathways such as DNA repair, efflux pump complexes, bacterial flagellar mobility, changes in metabolism or potential novel pathways may also play a role in temperature induced reduction in metronidazole resistance. Lastly, we confirmed that key resistance genes, *rdxA, frxA*, and *fdxB*, are not mutated by increased temperature conditions, suggesting that resistance changes can be attributed to gene expression or protein function changes. Further studies would be needed to elucidate these functional mechanisms in detail. Our approach of studying transcriptomic changes associated with temperature-related antibiotic resistance can highlight perturbed gene pathways as well as suggest candidate genes and pathways as potential novel drug targets for antimicrobials.

Our study examined the effect of 41°C temperature on *H. pylori* growth, metronidazole resistance and transcriptomic changes. Further studies are needed to investigate the detailed mechanisms driving reduced resistance to metronidazole. Effects at other temperatures as well as using strains with different profiles and mechanisms of resistance to various other antibiotics commonly used in *H. pylori* eradication could also be explored. Whether *in vitro* effects of increased temperature are also reflected *in vivo* remain to be elucidated.

At present, antibiotics treatment combined with proton-pump inhibitors is the main approach to treating *H. pylori* infections. Unfortunately, efficacy has decreased, due to the rapid rise in resistance rates (Fallone et al., 2019). Emergence of multi-drug resistance strains further exacerbates the problem (Boyanova et al., 2019). *H. pylori* colonizes and grows in gastric mucosa in humans which makes local treatment possible. Since the stomach mucosa of mammal species can withstand temperatures as high as 46°C (Kang et al., 2004), raising local temperatures have been postulated to help eradicate *H. pylori in vivo* complementing antibiotic-based therapies. One way to achieve this may be through well-controlled local photothermal/magnetic thermal treatments using nanomaterials which have shown early promise in suppressing bacterial growths (Mocan et al., 2017; Chen et al., 2018), including *H. pylori* (Wu et al., 2019). It is recently reported that gold nanostars@*H. pylori*-antibodies nanoprobes could target and kill *H. pylori* in the stomach in model animals under near-infrared laser irradiation (Zhi et al., 2019). However, feasibility and safety of sustained localized thermal treatment within human gastric mucosa need to be ascertained in a clinical setting in order for it to become a viable strategy for *H. pylori* eradication.

In conclusion, growth of *H. pylori* and resistance to metronidazole was significantly inhibited when cultured *in vitro* at 41°C. Transcriptomic analyses showed differential gene expression changes in more than one third of the genes with evidence of enrichment pointing to redox pathways as potential drivers of temperature related reduction in metronidazole resistance. Expression changes for genes in other resistance pathways may also play a role in temperature induced metronidazole sensitivity. Our findings suggest a novel approach in treating metronidazole and multi-drug resistant *H. pylori in vivo.*

## Data Availability Statement

The original contributions presented in the study are publicly available. This data can be found here: https://www.ncbi.nlm.nih.gov/sra/PRJNA718481.

## Author Contributions

MG carried out *H. pylori* growth experiments and antibiotics susceptibility testing, and drafted the manuscript. YH performed bioinformatic analysis and curation of related genes. XW participated in bioinformatic analysis. HT, FM, and BH participated in antibiotics susceptibility testing and gene expression analysis. BS and GW designed and conceived of the study, participated in its coordination, and wrote the manuscript. GW had primary responsibility for final content. All authors read and approved the final manuscript.

## Conflict of Interest

The authors declare that the research was conducted in the absence of any commercial or financial relationships that could be construed as a potential conflict of interest.

## Supplementary Material

Supplementary Figure 1 Agarose gel electrophoresis of gene fragments of *H. pylori* NCTC 11637 amplified by PCR. **(A)** Lanes 1–6, PCR fragments of gene *rdxA*; **(B)** Lane 1, PCR fragments of gene *frxA* of *H. pylori* NCTC 11637 cultured in 37°C; Lane 2, PCR fragments of gene *frxA* of *H. pylori* NCTC 11637 cultured in 41°C; Lanes 3–5, PCR fragments of gene *fdxB* of *H. pylori* NCTC 11637 cultured in 37°C with primer pairs 1–3, respectively, Lanes 6–8, PCR fragments of gene *fdxB* of *H. pylori* NCTC 11637 cultured in 41°C with primer pairs 1–3, respectively.

Supplementary Figure 2 The DNA sequence blast of *rdxA* gene in *H. pylori* 26695 and *H. pylori* NCTC 11637. The *H. pylori* 26695 is sensitive to metronidazole but *H. pylori* NCTC 11637 is resistant. Compared with *H. pylori* 26695, the structural variation of *rdxA* gene in *H. pylori* NCTC 11637 included point mutations and the insertion of mini-IS605, which caused *rdxA* inactivation and thus metronidazole resistance. 11637-*rdxA*-37: the *rdxA* gene sequence of *H. pylori* NCTC 11637 cultured in 37°C. 11637-*rdxA*-41: the *rdxA* gene sequence of *H. pylori* NCTC 11637 cultured in 41°C. 26695-*rdxA*: the *rdxA* gene reference sequence of *H. pylori* 26695. The dashed rectangle showed the start site of *rdxA* gene and the solid rectangle showed the termination site of *rdxA* gene. The figure showed that the 11637-*rdxA*-37 and 11637-*rdxA*-41 were exactly the same. The high light region showed the insertion of mini-IS605, one of the endogenous transposable elements, in *H. pylori* NCTC 11637.

Supplementary Figure 3 The DNA sequence blast of *frxA* gene in *H. pylori* 26695 and *H. pylori* NCTC 11637. 11637-*frxA*-37: the *frxA* gene sequence of *H. pylori* NCTC 11637 cultured in 37°C. 11637-*frxA*-41: the *frxA* gene sequence of *H. pylori* NCTC 11637 cultured in 41°C. 26695-*frxA*: the *frxA* gene reference sequence of *H. pylori* 26695. The figure showed that the 11637-*frxA*-37 and 11637-*frxA*-41 were exactly the same.

Supplementary Figure 4 The DNA sequence blast of *fdxB* gene in *H. pylori* 26695 and *H. pylori* NCTC 11637. 11637-*fdxB*-37: the *fdxB* gene sequence of *H. pylori* NCTC 11637 cultured in 37°C. 11637-*fdxB*-41: the *fdxB* gene sequence of *H. pylori* NCTC 11637 cultured in 41°C. 26695-*fdxB*: the *fdxB* gene reference sequence of *H. pylori* 26695. The figure showed that the 11637-*fdxB*-37 and 11637-*fdxB*-41 were exactly the same.

## Abbreviations

BP: biological process
CC: cellular component
CFU: colony forming units
DEGs: differentially expressed genes
*E*-test: Epsilometer test
GC: gastric cancer
GO: Gene Ontology
*H. pylori*: *Helicobacter pylori*
KEGG: Kyoto Encyclopedia of Genes and Genomes
MF: molecular function
MIC: minimum inhibitory concentration
PBS: phosphate-buffered saline
PCR: polymerase chain reaction
Pfam: protein families database.
