# Supplementary figures and images for: Effect of Temperature on Metronidazole Resistance in Helicobacter pylori
Source: Front Microbiol. 2021 May 19;12:681911. doi: 10.3389/fmicb.2021.681911 (PMC8170400; doi:10.3389/fmicb.2021.681911)

M 1 2 3 4 5 6

**A**

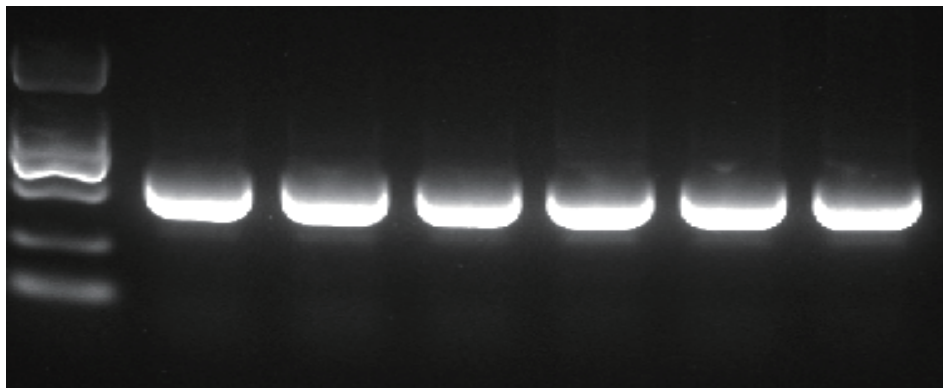

1 2 M 3 4 5 6 7 8

**B**

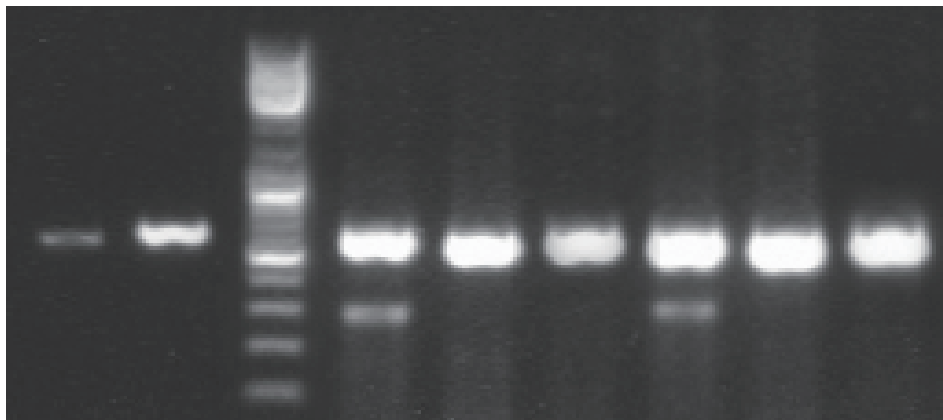

Supplement: Supplementary Figure 1 — Agarose gel electrophoresis of gene fragments of H. pylori NCTC 11637 amplified by PCR. (A) Lanes 1–6, PCR fragments of gene rdxA; (B) Lane 1, PCR fragments of gene frxA of H. pylori NCTC 11637 cultured in 37°C; Lane 2, PCR fragments of gene frxA of H. pylori NCTC 11637 cultured in 41°C; Lanes 3–5, PCR fragments of gene fdxB of H. pylori NCTC 11637 cultured in 37°C with primer pairs 1–3, respectively, Lanes 6–8, PCR fragments of gene fdxB of H. pylori NCTC 11637 cultured in 41°C with primer pairs 1–3, respectively. [file Data_Sheet_1.PDF]
