# Supplementary material for: Effect of Temperature on Metronidazole Resistance in Helicobacter pylori
Source: Front Microbiol. 2021 May 19;12:681911. doi: 10.3389/fmicb.2021.681911 (PMC8170400; doi:10.3389/fmicb.2021.681911)
Supplement: Supplementary Figure 2 — The DNA sequence blast of rdxA gene in H. pylori 26695 and H. pylori NCTC 11637. The H. pylori 26695 is sensitive to metronidazole but H. pylori NCTC 11637 is resistant. Compared with H. pylori 26695, the structural variation of rdxA gene in H. pylori NCTC 11637 included point mutations and the insertion of mini-IS605, which caused rdxA inactivation and thus metronidazole resistance. 11637-rdxA-37: the rdxA gene sequence of H. pylori NCTC 11637 cultured in 37°C. 11637-rdxA-41: the rdxA gene sequence of H. pylori NCTC 11637 cultured in 41°C. 26695-rdxA: the rdxA gene reference sequence of H. pylori 26695. The dashed rectangle showed the start site of rdxA gene and the solid rectangle showed the termination site of rdxA gene. The figure showed that the 11637-rdxA-37 and 11637-rdxA-41 were exactly the same. The high light region showed the insertion of mini-IS605, one of the endogenous transposable elements, in H. pylori NCTC 11637. [file Data_Sheet_2.PDF]

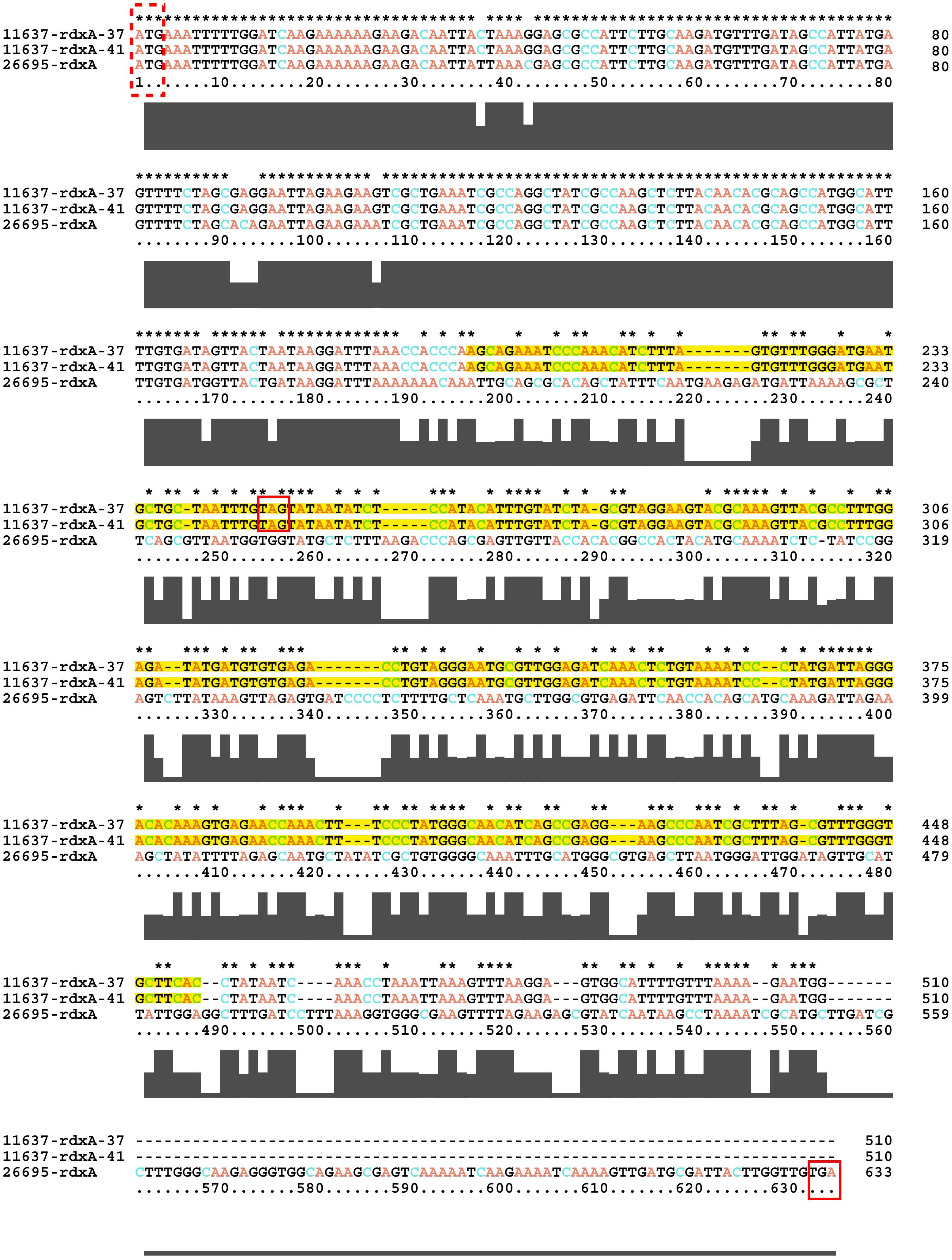

11637-rdxA-37 ATGAAATTTTTGGATCAAGAAAAAAGAAGACAATTACTAAAGGAGCGCCATTCTTGCAAGATGTTTGATAGCCATTATGA 80
11637-rdxA-41 ATGAAATTTTTGGATCAAGAAAAAAGAAGACAATTACTAAAGGAGCGCCATTCTTGCAAGATGTTTGATAGCCATTATGA 80
26695-rdxA ATGAAATTTTTGGATCAAGAAAAAAGAAGACAATTATTAAACGAGCGCCATTCTTGCAAGATGTTTGATAGCCATTATGA 80
1.......10.........20.........30.........40.........50.........60.........70.........80
11637-rdxA-37 GTTTTCTAGCGAGGAATTAGAAGAAGTCGCTGAAATCGCCAGGCTATCGCCAAGCTCTTACAACACGCAGCCATGGCATT 160
11637-rdxA-41 GTTTTCTAGCGAGGAATTAGAAGAAGTCGCTGAAATCGCCAGGCTATCGCCAAGCTCTTACAACACGCAGCCATGGCATT 160
26695-rdxA GTTTTCTAGCACAGAATTAGAAGAAATCGCTGAAATCGCCAGGCTATCGCCAAGCTCTTACAACACGCAGCCATGGCATT 160
........90.......100.......110.......120.......130.......140.......150.......160
11637-rdxA-37 TTGTGATAGTTACTAATAAGGATTTAAACCACCCAAGCAGAAATCCCAAACATCTTTA-------GTGTTTGGGATGAAT 233
11637-rdxA-41 TTGTGATAGTTACTAATAAGGATTTAAACCACCCAAGCAGAAATCCCAAACATCTTTA-------GTGTTTGGGATGAAT 233
26695-rdxA TTGTGATGGTTACTGATAAGGATTTAAAAAAACAAATTGCAGCGCACAGCTATTTCAATGAAGAGATGATTAAAAGCGCT 240
.......170.......180.......190.......200.......210.......220.......230.......240
11637-rdxA-37 GCTGC-TAATTTGTAGTATAATATCT-----CCATACATTTGTATCTA-GCGTAGGAAGTACGCAAAGTTACGCCTTTGG 306
11637-rdxA-41 GCTGC-TAATTTGTAGTATAATATCT-----CCATACATTTGTATCTA-GCGTAGGAAGTACGCAAAGTTACGCCTTTGG 306
26695-rdxA TCAGCGTTAATGGTGGTATGCTCTTTAAGACCCAGCGAGTTGTTACCACACGGCCACTACATGCAAAATCTC-TATCCGG 319
.......250.......260.......270.......280.......290.......300.......310.......320
11637-rdxA-37 AGA--TATGATGTGTGAGA-------CCTGTAGGGAATGCGTTGGAGATCAAACTCTGTAAAATCC--CTATGATTAGGG 375
11637-rdxA-41 AGA--TATGATGTGTGAGA-------CCTGTAGGGAATGCGTTGGAGATCAAACTCTGTAAAATCC--CTATGATTAGGG 375
26695-rdxA AGTCTTATAAAGTTAGAGTGATCCCCTCTTTTGCTCAAATGCTTGGCGTGAGATTCAACCACAGCATGCAAAGATTAGAA 399
.......330.......340.......350.......360.......370.......380.......390.......400
11637-rdxA-37 ACACAAAGTGAGAACCAAACTT---TCCCTATGGGCAACATCAGCCGAGG---AAGCCCAATCGCTTTAG-CGTTTGGGT 448
11637-rdxA-41 ACACAAAGTGAGAACCAAACTT---TCCCTATGGGCAACATCAGCCGAGG---AAGCCCAATCGCTTTAG-CGTTTGGGT 448
26695-rdxA AGCTATATTTTAGAGCAATGCTATATCGCTGTGGGGCAAATTTGCATGGGCGTGAGCTTAATGGGATTGGATAGTTGCAT 479
.......410.......420.......430.......440.......450.......460.......470.......480
11637-rdxA-37 GCTTCAC--CTATAATC----AAACCTAAATTAAAGTTTAAGGA---GTGGCATTTTGTTTAAAA--GAATGG------- 510
11637-rdxA-41 GCTTCAC--CTATAATC----AAACCTAAATTAAAGTTTAAGGA---GTGGCATTTTGTTTAAAA--GAATGG------- 510
26695-rdxA TATTGGAGGCTTTGATCCTTTAAAGGTGGGCGAAGTTTTAGAAGAGCGTATCAATAAGCCTAAAATCGCATGCTTGATCG 559
.......490.......500.......510.......520.......530.......540.......550.......560
11637-rdxA-37 --- 510
11637-rdxA-41 --- 510
26695-rdxA CTTTGGGCAAGAGGGTGGCAGAAGCGAGTCAAAAATCAAGAAAATCAAAAGTTGATGCGATTACTTGGTTGTGA 633
.......570.......580.......590.......600.......610.......620.......630...
