# Supplementary material for: Effect of Temperature on Metronidazole Resistance in Helicobacter pylori
Source: Front Microbiol. 2021 May 19;12:681911. doi: 10.3389/fmicb.2021.681911 (PMC8170400; doi:10.3389/fmicb.2021.681911)
Supplement: Supplementary Figure 3 — The DNA sequence blast of frxA gene in H. pylori 26695 and H. pylori NCTC 11637. 11637-frxA-37: the frxA gene sequence of H. pylori NCTC 11637 cultured in 37°C. 11637-frxA-41: the frxA gene sequence of H. pylori NCTC 11637 cultured in 41°C. 26695-frxA: the frxA gene reference sequence of H. pylori 26695. The figure showed that the 11637-frxA-37 and 11637-frxA-41 were exactly the same. [file Data_Sheet_3.PDF]

```
               ***************************************************** ******** ***** ***********
frxA-11637-37 ATGGACAGAGAACAAGTGGTTGCTTTACAGCACCAACGATTTGCTGCAAAAAA-TACGATCCCAATCGCCGTATTTCCCA    79
frxA-11637-41 ATGGACAGAGAACAAGTGGTTGCTTTACAGCACCAACGATTTGCTGCAAAAAA-TACGATCCCAATCGCCGTATTTCCCA    79
frxA-26695    ATGGACAGAGAACAAGTGGTTGCTTTACAGCACCAACGATTTGCTGCAAAAAAATACGATCCTAATCGTCGTATTTCCCA    80

               *** ***************************** ************************** *******************
frxA-11637-37 AAAAGATTGGGAAGCTTTGGTTGAAGTGGGGAGGTTAGCCCCTTCTTCAATCGGGCTTGAGCCATGGAAAATGCTTTTAT   159
frxA-11637-41 AAAAGATTGGGAAGCTTTGGTTGAAGTGGGGAGGTTAGCCCCTTCTTCAATCGGGCTTGAGCCATGGAAAATGCTTTTAT   159
frxA-26695    AAAGGATTGGGAAGCTTTGGTTGAAGTGGGGAGATTAGCCCCTTCTTCAATCGGGCTTGAACCATGGAAAATGCTTTTAT   160

               * ** ********************************************* *********** *********** ******
frxA-11637-37 TAAAGAATGAACGCATGAAAGAAGATTTAAAACCGATGGCCTGGGGGGCACTTTTTGGTTTAGAGGGAGCGAGTCATTTT   239
frxA-11637-41 TAAAGAATGAACGCATGAAAGAAGATTTAAAACCGATGGCCTGGGGGGCACTTTTTGGTTTAGAGGGAGCGAGTCATTTT   239
frxA-26695    TGAAAAATGAACGCATGAAAGAAGATTTAAAACCGATGGCCTGGGGGGCGCTTTTTGGTTTGGAGGGAGCGAGCCATTTT   240

               ************************************************** *****************************
frxA-11637-37 GTCATTTATCTTGCGCGAAAAGGCGTTACTTATGACAGCGATTACGTTAAGAAAGTGATGCATGAGGTTAAAAAAAGGGA   319
frxA-11637-41 GTCATTTATCTTGCGCGAAAAGGCGTTACTTATGACAGCGATTACGTTAAGAAAGTGATGCATGAGGTTAAAAAAAGGGA   319
frxA-26695    GTCATTTATCTTGCGCGAAAAGGCGTTACTTATGACAGCGATTACGTTAAAAAAGTGATGCATGAGGTTAAAAAAAGGGA   320

               *************** ************************************************************** *
frxA-11637-37 TTATGACACTAATTCCAGGTTCGCTCAAATCATCAAAAATTTCCAAGAGAACGATATGAAACTCAATAGCGAACGATCCT   399
frxA-11637-41 TTATGACACTAATTCCAGGTTCGCTCAAATCATCAAAAATTTCCAAGAGAACGATATGAAACTCAATAGCGAACGATCCT   399
frxA-26695    TTATGACACTAATTCTAGGTTCGCTCAAATCATCAAAAATTTCCAAGAGAACGATATGAAACTCAATAGCGAACGATCTT   400

               ********************************************************************************
frxA-11637-37 TGTTTGATTGGGCTAGCAAGCAGACTTATATCCAAATGGCGAACATGATGATGGCAGCGGCCATGTTAGGGATTGATTCT   478
frxA-11637-41 TGTTTGATTGGGCTAGCAAGCAGACTTATATCCAAATGGCGAACATGATGATGGCAGCGGCCATGTTAGGGATTGATTCT   479
frxA-26695    TGTTTGATTGGGCTAGCAAGCAGACTTATATCCAAATGGCGAACATGATGATGGCAGCGGCCATGTTAGGGATTGATTCT   480

               ********************************************************************************
frxA-11637-37 TGCCCGATTGAAGGGTATGATCAAGAAAAAGTGGAGGCTTATTTAGAGGAAAAAGGCTATCTGAACACGGCGGAATTTGG   558
frxA-11637-41 TGCCCGATTGAAGGGTATGATCAAGAAAAAGTGGAGGCTTATTTAGAGGAAAAAGGCTATCTGAACACGGCGGAATTTGG   559
frxA-26695    TGCCCGATTGAAGGGTATGATCAAGAAAAAGTGGAGGCTTATTTAGAGGAAAAAGGCTATCTGAACACGGCGGAATTTGG   560

               ****************************** ************************** **********************
frxA-11637-37 CGTGTCGGTAATGGCTTGTTTTGGTTATCGCAACCAAGAAATCACCCCTAAAACCCGTTGGAAGACAGAAGTTATTTATG   638
frxA-11637-41 CGTGTCGGTAATGGCTTGTTTTGGTTATCGCAACCAAGAAATCACCCCTAAAACCCGTTGGAAGACAGAAGTTATTTATG   639
frxA-26695    CGTGTCGGTAATGGCTTGTTTTGGTTATCGTAACCAAGAAATCACCCCTAAAACCCGCTGGAAGACAGAAGTTATTTATG   640

               **************
frxA-11637-37 AAGTGATTGAATAA   652
frxA-11637-41 AAGTGATTGAATAA   653
frxA-26695    AAGTGATTGAATAA   654
```
