# Supplementary material for: Effect of Temperature on Metronidazole Resistance in Helicobacter pylori
Source: Front Microbiol. 2021 May 19;12:681911. doi: 10.3389/fmicb.2021.681911 (PMC8170400; doi:10.3389/fmicb.2021.681911)
Supplement: Supplementary Figure 4 — The DNA sequence blast of fdxB gene in H. pylori 26695 and H. pylori NCTC 11637. 11637-fdxB-37: the fdxB gene sequence of H. pylori NCTC 11637 cultured in 37°C. 11637-fdxB-41: the fdxB gene sequence of H. pylori NCTC 11637 cultured in 41°C. 26695-fdxB: the fdxB gene reference sequence of H. pylori 26695. The figure showed that the 11637-fdxB-37 and 11637-fdxB-41 were exactly the same. [file Data_Sheet_4.PDF]

```
               *********************************** ****************************************** *
fdxB-11637-37  ATGCTTGAAACTTCTAGCCATTTTTTAAAATCGTTCCGCTTGAAGCGTTATATAGGGTTTTTATTGATTTCTTTAGCGCT   80
fdxB-11637-41  ATGCTTGAAACTTCTAGCCATTTTTTAAAATCGTTCCGCTTGAAGCGTTATATAGGGTTTTTATTGATTTCTTTAGCGCT   80
fdxB-26695     ATGCTTGAAACTTCTAGCCATTTTTTAAAATCGTTTCGCTTGAAGCGTTATATAGGGTTTTTATTGATTTCTTTAGCGTT   80

               * ** ********************************** *********** ****************************
fdxB-11637-37  ACTAGTCACGCCCTTTGTTCGCATTGATGGGGCGCATTTATTTTTGATCTCCTTTGAGCATAAGCAACTGCATTTTTTAG   160
fdxB-11637-41  ACTAGTCACGCCCTTTGTTCGCATTGATGGGGCGCATTTATTTTTGATCTCCTTTGAGCATAAGCAACTGCATTTTTTAG   160
fdxB-26695     ATTAATCACGCCCTTTGTTCGCATTGATGGGGCGCATTTGTTTTTGATCTCTTTTGAGCATAAGCAACTGCATTTTTTAG   160

               ********************************************************************************
fdxB-11637-37  GCAAGATCTTTAGCGCTGAAGAATTGCAAGTCATGCCTTTTATGGTTATTTTGCTTTTTATAGGGATTTTTTTCATCACC   240
fdxB-11637-41  GCAAGATCTTTAGCGCTGAAGAATTGCAAGTCATGCCTTTTATGGTTATTTTGCTTTTTATAGGGATTTTTTTCATCACC   240
fdxB-26695     GCAAGATCTTTAGCGCTGAAGAATTGCAAGTCATGCCTTTTATGGTTATTTTGCTTTTTATAGGGATTTTTTTCATCACC   240

               ************************** *****************************************************
fdxB-11637-37  ACTAGCCTTGGGCGTGTGTGGTGCGGGTGGGCTTGCCCGCAAACCTTTTTAAGGGTGCTTTATAGAGATGTGATTGAAAC   320
fdxB-11637-41  ACTAGCCTTGGGCGTGTGTGGTGCGGGTGGGCTTGCCCGCAAACCTTTTTAAGGGTGCTTTATAGAGATGTGATTGAAAC   320
fdxB-26695     ACTAGCCTTGGGCGTGTGTGGTGCGGTTGGGCTTGCCCGCAAACCTTTTTAAGGGTGCTTTATAGAGATGTGATTGAAAC   320

               ******************************************** ********************* *************
fdxB-11637-37  CAAGATTTTCAAACTCCATAAAAAGATCAGCAACAAGCAAGAAAACCCTAAAAACACCCCAAGCTATAAGATCCGTAAAG   400
fdxB-11637-41  CAAGATTTTCAAACTCCATAAAAAGATCAGCAACAAGCAAGAAAACCCTAAAAACACCCCAAGCTATAAGATCCGTAAAG   400
fdxB-26695     CAAGATTTTCAAACTCCATAAAAAGATCAGCAACAAGCAAGAAAGCCCTAAAAACACCCCAAGCTACAAGATCCGTAAAG   400

                 ******************************** ******************************************** 
fdxB-11637-37  CGTTGAGCGTTTTATTGTTCGCTCCTGTTGTGGCAGGGCTAATGATGTTGTTTTTCTTTTATTTCATCGCCCCAGAAGAC   480
fdxB-11637-41  CGTTGAGCGTTTTATTGTTCGCTCCTGTTGTGGCAGGGCTAATGATGTTGTTTTTCTTTTATTTCATCGCCCCAGAAGAC   480
fdxB-26695     TATTGAGCGTTTTATTGTTCGCTCCTGTTGTGGCGGGGCTAATGATGTTGTTTTTCTTTTATTTCATCGCCCCAGAAGAT   480

               ************************************ ************************************* *****
fdxB-11637-37  TTTTTTATGTATCTTAAAAACCCTAGCGATCACCCTGTTGCTATGGGTTTTTGGCTTTTTAGCACGGCTGTGGTACTATT   560
fdxB-11637-41  TTTTTTATGTATCTTAAAAACCCTAGCGATCACCCTGTTGCTATGGGTTTTTGGCTTTTTAGCACGGCTGTGGTACTATT   560
fdxB-26695     TTTTTTATGTATCTTAAAAACCCTAGCGATCACCCTATTGCTATGGGTTTTTGGCTTTTTAGCACGGCTGTGGTGCTATT   560

               ********************************************************************************
fdxB-11637-37  TGATATAGTGGTGGTTGCGGAGCGTTTTTGCATTTATTTATGCCCTTACGCTAGGGTGCAATCGGTGTTGTATGACAATG   640
fdxB-11637-41  TGATATAGTGGTGGTTGCGGAGCGTTTTTGCATTTATTTATGCCCTTACGCTAGGGTGCAATCGGTGTTGTATGACAATG   640
fdxB-26695     TGATATAGTGGTGGTTGCGGAGCGTTTTTGCATTTATTTATGCCCTTACGCTAGGGTGCAATCGGTGTTGTATGACAATG   640

               * *********** ****************** ******************************************* ***
fdxB-11637-37  ATACCTTAAACCCCATTTATGATGAAAAGCGTGGCGGAGCGCTTTATAATAATCAGGGCCATCTCTTCCCCTTACCCCCC   720
fdxB-11637-41  ATACCTTAAACCCCATTTATGATGAAAAGCGTGGCGGAGCGCTTTATAATAATCAGGGCCATCTCTTCCCCTTACCCCCC   720
fdxB-26695     ACACCTTAAACCCTATTTATGATGAAAAGCGCGGCGGAGCGCTTTATAATAATCAGGGCCATCTCTTCCCCTTACCTCCC   720

               ***********   ********************* ********************************************
fdxB-11637-37  AAAAAACGCAGTGTAGAAAACGAATGCGTGAATTGCTTGCATTGCGTGCAGGTTTGCCCCACGCATATTGACATCAGGAA   800
fdxB-11637-41  AAAAAACGCAGTGTAGAAAACGAATGCGTGAATTGCTTGCATTGCGTGCAGGTTTGCCCCACGCATATTGACATCAGGAA   800
fdxB-26695     AAAAAACGCAGCCCAGAAAACGAATGCGTGAATTGTTTGCATTGCGTGCAGGTTTGCCCCACGCATATTGACATCAGGAA   800
```

```
              *************************** **************************************** **********
fdxB-11637-37 GGGCTTGCAATTAGAATGCATCAATTGCTTAGAATGCGTGGATGCATGCACGATTACCATGGCTAAATACAACCGCCCTT   880
fdxB-11637-41 GGGCTTGCAATTAGAATGCATCAATTGCTTAGAATGCGTGGATGCATGCACGATTACCATGGCTAAATACAACCGCCCTT   880
fdxB-26695    GGGCTTGCAATTAGAATGCATCAATTGTTTAGAATGCGTGGATGCATGCACGATTACCATGGCTAAATTTAACCGCCCTT   880

              ********************** ***** ***** **************** *** ***********************
fdxB-11637-37 CACTCATCCAATGGTCTTCAACCAACGCCATTAACACGCGCCAAAAAGTGCGCCTAGTGCGTTTAAAAACGATCGCTTAT   960
fdxB-11637-41 CACTCATCCAATGGTCTTCAACCAACGCCATTAACACGCGCCAAAAAGTGCGCCTAGTGCGTTTAAAAACGATCGCTTAT   960
fdxB-26695    CACTCATCCAATGGTCTTCAACTAACGCTATTAATACGCGCCAAAAAGTGCACCTGGTGCGTTTAAAAACGATCGCTTAC   960

               ************** ******* ********************************************************
fdxB-11637-37 TTGGGGGTTATCGCTGTTGTGATAGCTCTTTTAGCCATCACTTCGTTTAAAAAAGAACGCATGCTCTTAGACATTAACCG  1040
fdxB-11637-41 TTGGGGGTTATCGCTGTTGTGATAGCTCTTTTAGCCATCACTTCGTTTAAAAAAGAACGCATGCTCTTAGACATTAACCG  1040
fdxB-26695    ATGGGGGTTATCGCTATTGTGATCGCTCTTTTAGCCATCACTTCGTTTAAAAAAGAACGCATGCTCTTAGACATTAACCG  1040

              ****************************** *************************************************
fdxB-11637-37 CAACAGCGATCTGTATGAATTGCGCTCTAGTGGGTATGTGGATAACGATTACGTGTTTTTATTCCACAACACGGACAATA  1120
fdxB-11637-41 CAACAGCGATCTGTATGAATTGCGCTCTAGTGGGTATGTGGATAACGATTACGTGTTTTTATTCCACAACACGGACAATA  1120
fdxB-26695    CAACAGCGATCTGTATGAATTGCGCTCTAGCGGGTATGTGGATAACGATTACGTGTTTTTATTCCACAACACGGACAATA  1120

              *********************** ******************* ** ***************** ***************
fdxB-11637-37 AAGACCATGAGTTTTATTTCAAAATTTTAGGGCAAAAAGACATCCAAATCAAAAAGCCTTTAAACCCTATCGCCATTAAA  1200
fdxB-11637-41 AAGACCATGAGTTTTATTTCAAAATTTTAGGGCAAAAAGACATCCAAATCAAAAAGCCTTTAAACCCTATCGCCATTAAA  1200
fdxB-26695    AAGACCATGAGTTTTATTTCAAAGTTTTAGGGCAAAAAGACATTCAGATCAAAAAGCCTTTAAATCCTATCGCCATTAAA  1200

              ********************************* ************************** *******************
fdxB-11637-37 GCCGGGCAAAAGATTAAAGCGGTAGTGATTTTACGAAAACCCCTAAAGAGTAACGCCACAAAATACAAGAACGCTAAAGA  1280
fdxB-11637-41 GCCGGGCAAAAGATTAAAGCGGTAGTGATTTTACGAAAACCCCTAAAGAGTAACGCCACAAAATACAAGAACGCTAAAGA  1280
fdxB-26695    GCCGGGCAAAAGATTAAAGCGGTAGTGATTTTAAGAAAACCCCTAAAGAGTAACGCCACAGAATACAAGAACGCTAAAGA  1280

              ****** ***** *********************** *******************************************
fdxB-11637-37 CGCTCTCATCCCTATTACCATACAAGCTTATAGCGCAGACGATAAGAATATTACGATAGAAAGGGAATCGGTGTTTATTG  1360
fdxB-11637-41 CGCTCTCATCCCTATTACCATACAAGCTTATAGCGCAGACGATAAGAATATTACGATAGAAAGGGAATCGGTGTTTATTG  1360
fdxB-26695    CGCTCTAATCCCCATTACCATACAAGCTTATAGCGCGGACGATAAGAATATTACGATAGAAAGGGAATCGGTGTTTATTG  1360

              *****************
fdxB-11637-37 CACCAAGTGAGGATTGA  1377
fdxB-11637-41 CACCAAGTGAGGATTGA  1377
fdxB-26695    CACCAAGTGAGGATTGA  1377
```
